# Preparation and Characterization of Prickly Ash Peel Oleoresin Microcapsules and Flavor Retention Analysis

**DOI:** 10.3390/foods13111726

**Published:** 2024-05-31

**Authors:** Zhiran Zhang, Ziyan Zhang, Xichao Li, Sen Zhou, Mengkai Liu, Shengxin Li, He Liu, Hui Gao, Aiyun Zhao, Yongchang Zhang, Liu Huang, Jie Sun

**Affiliations:** 1 College of Life Sciences, Qingdao University, Qingdao 266071, China; zzrzgj@163.com (Z.Z.); zhangziyan77@126.com (Z.Z.); jason_zhousen@163.com (S.Z.); liumengkaikai@163.com (M.L.); lsxy0215@163.com (S.L.); heliu1997@126.com (H.L.); zhaoaiyun@qdu.edu.cn (A.Z.); 2 National Engineering Research Centre for Intelligent Electrical Vehicle Power System (Qingdao), College of Mechanical & Electronic Engineering, Qingdao University, Qingdao 266071, China; lixichao@qdu.edu.cn; 3 LIHOOS (Qingdao) Food Co., Ltd., Qingdao 266000, China; lihezyc@lihoos.com (Y.Z.); lihehl@lihoos.com (L.H.)

**Keywords:** Prickly ash peel oleoresin, microcapsule, spray-drying, freeze-drying, flavor substance

## Abstract

Prickly ash peel oleoresin (PPO) is a highly concentrated oil of Prickly ash essential oil and has a stronger aroma. However, its low water solubility, high volatility, difficulty in transport and storage, and decomposition by light, heat, and oxygen limit its wider application. To solve this problem, this study used freeze-drying or spray-drying, with soybean protein isolate (SPI) or gum Arabic (GA), combined with aqueous maltodextrin (MD) as the encapsulating agents to prepare four types of PPO microcapsules (POMs). Spray-dried microcapsules with GA as the encapsulating agent achieved a high encapsulation efficiency (EE) of 92.31 ± 0.31%, improved the thermal stability of the PPO, and had spherical morphology. (Headspace solid-phase microextraction/gas chromatography–mass spectrometry) HS-SPME/GC-MS detected 41 volatile compounds in PPO; of these, linalool, β-myrcene, sabinene, and D-limonene were identified as key flavor components. Principal component analysis (PCA) effectively distinguished the significant differences in flavor between PPO, spray-dried SPI/MD microcapsules (SS), and spray-dried GA/MD microcapsules (SG). During 15 days of air-exposure, the loss of flavor from SG (54.62 ± 0.54%) was significantly lower than PPO (79.45 ± 1.45%) and SS (57.55 ± 0.36%). During the air-exposure period, SG consistently had the highest antioxidant capacity, making it desirable for PPO packaging, and expanding its potential applications within the food industry.

## 1. Introduction

Prickly ash (*Zanthoxylum bungeanum*) is a deciduous shrub, or small tree, from the Rutaceae family and the *Zanthoxylum* genus [1]. It is widespread in tropical and subtropical regions, including Asia, America, Africa, and Oceania [2]. Prickly ash is a popular spice that enriches the flavor and color of food and masks unpleasant odors. Prickly ash peel oleoresin (PPO) is the liquid concentrated essential oil extracted from prickly ash using organic, or super/subcritical solvents, which has a much stronger aroma and flavor than the original plant material, as well as medicinally useful biological activities [3,4]. Studies suggest that flavors, such as linalool, D-limonene, α-pinene, and β-myrcene, found in PPO not only significantly contribute to the presentation of woody, balsamic, and other flavors but also have biological activities, such as antioxidant and antibacterial [3,5,6,7]. However, the quality, sensory attributes, and flavor compound composition of PPO degrade during transportation and storage. The flavor compound content of PPO decreased at temperatures above 70 °C [8] and some key flavors degraded, increasing the bitterness of PPO. The volatility and instability of these compounds restricts the potential applications of PPO. Microencapsulation is the conversion of essential oils into solid powders by embedding, which is a good potential approach for stabilizing and extending the shelf-life of oils and can reduce the formation of unpleasant flavors and odors, maintain biological activity, and expand the potential applications of PPO.

Microencapsulation is a technique for packing a solid, liquid, or gaseous material into a sealed microcapsule and releasing its contents at a controlled rate under specific conditions [9]. Microencapsulation can effectively encapsulate flavor, stabilize the activity of sensitive compounds, such as vitamins and probiotics, against degradation, and maintain antioxidant capacity [10]. In recent years, microencapsulation of various plant-derived essential oils, such as lemon essential oil and sea buckthorn seed oil, has been used to stabilize their main components and extend their shelf-life [11,12].

Various methods have been devised to prepare microcapsules, the most common of which are spray-drying and freeze-drying. Spray drying has a low cost for mass production and produces powders with excellent physicochemical properties, that are suitable for the rapid production of encapsulated flavor and essential oils [13,14]. Freeze-drying needs much less water and is more suitable for microencapsulation of thermally unstable bioactive compounds [15], but is time-consuming and expensive, so it is not suitable for industrial production [16]. However, a systematic comparison between the two preparation methods has not been conducted for POM.

The encapsulation efficiency (EE) achieved depends not only on the preparation method but also on the choice of encapsulating agent. Polysaccharides are the most widely used encapsulation materials for food use, of which maltodextrin (MD) has the advantages of high water solubility, neutral aroma and taste, low viscosity, and good oxidative stability in the presence of oxidizing encapsulated materials [10,17]. However, MD sometimes has a suboptimal emulsification capacity and limited oil retention capacity, necessitating combination with other materials to enhance the EE [18]. Gum Arabic (GA) is a colorless plant polysaccharide exuded from acacia trees, which has good biocompatibility and biodegradability and is widely used in the food industry as an amphiphilic polysaccharide [19]. Soy protein isolate (SPI) is a major source of plant protein, because it has superior protein quality to those of other plant proteins, beneficial health effects, and can interact with small bioactive molecules through hydrophobic interactions, hydrogen bonds, and disulfide bonds [20]. MD, GA, and SPI are all suitable as encapsulation materials, because of their good emulsification and film-forming properties [18,21]. However, few studies have been conducted on the complexation behavior of the GA, SPI, and MD as encapsulating materials for microencapsulation, particularly with PPO.

HS-SPME/GC-MS is a common technique for the analysis of food flavor. It enables quantitative analyses of the relative levels of volatile compounds [21]. The odor activity value (OAV) is the ratio of the concentration of a volatile organic compound (VOC) to its odor detection threshold, and when combined with GC-MS quantification, can assess the impact of individual VOCs on food aromas and identify the critical aroma compounds. This approach has previously been applied to evaluate VOCs in diverse food products, such as walnut oil, olive oil, and other plant essential oils [21,22]. In this study, the flavor composition and content of POM were analyzed using HS-SPME/GC-MS technology during different air-exposure periods, while also evaluating their ability to retain flavors.

**Figure 1:**
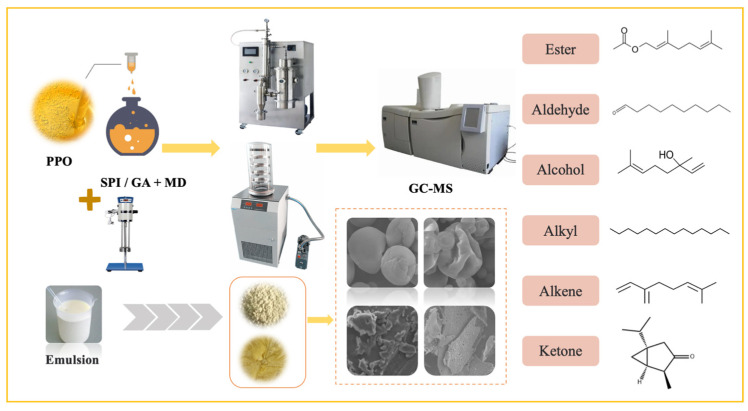
Preparation and physicochemical property analysis of PPO microcapsules.

The preparation, performance characterization, and flavor retention capacity of POMs have not been comprehensively and systematically investigated. In this study, POMs were prepared using spray-drying or freeze-drying. The physicochemical properties of the POMs were analyzed by combining particle size analysis, Fourier transform infrared (FTIR) spectroscopy, differential scanning calorimetry (DSC), and scanning electron microscopy (SEM). Changes in volatile flavor during air exposure were determined by HS-SPME/GC-MS (Figure 1). These findings effectively improved the flavor retention effect of PPO, provided valuable insights for future research on POMs, and have the potential to enhance their application in foods.

## 2. Materials and Methods

SPI, GA, and MD were from Shengda Food Technology Co., Ltd. (Longhai, China) and PPO (volatile oil 50 mL/100 g) was from the LIHOOS (Qingdao) Food Co., Ltd. (Qingdao, China). Ethanol was from Merck (Chengdu, China), and petroleum ether, 1, 1-diphenyl-2-picrylhydrazil (DPPH), FeCl3, salicylic acid, ascorbic acid, lecithin, and H_2_O_2_ were all of analytical grade from Macklin (Shanghai, China). n-Alkane GC reference standards (C7–C30) were from TCI (Beijing, China). Headspace glass vials were purchased from Sigma-Aldrich (St. Louis, MO, USA).

### 2.1. Emulsion Preparation

Two encapsulating agent emulsions were prepared for PPO encapsulation. They consisted of SPI (1.5% *w*/*w*) or GA (1.5% *w*/*w*), combined with MD (3.5% *w*/*w*), which were magnetically stirred until homogeneity [23,24]. Subsequently, PPO (oil:encapsulating agent = 1:4) was added, followed by homogenization at 12000 rpm/min for 5 min using a shear homogenizer (Ultra Turrax T-25, IKA, Stauffen, Germany).

### 2.2. Preparation of Microcapsules

The emulsions were converted into powder form using freeze-drying and spray-drying. Spray-drying used a laboratory spray-dryer (Buchi B-290, Buchi Corporation, Shanghai, China) at an inlet temperature of 130 °C and an outlet temperature of 60 ± 2 °C. The sample entry temperature was maintained at 25.0 ± 0.5 °C with a flow rate of 8 mL/min, and the outlet air temperature was maintained at 80 ± 2 °C.

The freeze-drying method was as follows: the PPO emulsion was frozen at −20 °C for 24 h, then dried using an Alpha 1–4 LD Plus model freeze-dryer (Martin Christ, Osterode am Harz, Germany) at 0.8 Pa and −60 °C for 48 h. The spray-dried microcapsules were designated as SS (SPI and MD) and SG (GA and MD), and the corresponding freeze-dried microcapsules as FS and FG, respectively.

### 2.3. Microcapsule Characterization

#### 2.3.1. Particle Size Analysis

Here, 0.2 g microcapsules (FS, FG, SS, and SG) were dissolved in deionized water (2 mL), centrifuged at 1611 g (4000 rpm in M1650 rotor, Merrick Instruments Co., Ltd., Shanghai, China) for 2 min to remove insoluble impurities and the supernatant used for particle size determination, using a Zetasizer laser diffraction particle size analyzer (Malvern, UK). Each measurement was repeated three times.

#### 2.3.2. Encapsulation Efficiency (EE) Determination

EE was determined as described in [18]. Before each experiment, the weight of the container without the sample needs to be constant, and the specific method was determined as follows: The glass beaker was dried at 80 °C in the blast drying oven (LICHEN, 101-00BS, Shanghai, China) for 1 h and then transferred to the glass desiccator (KESD, Shenzhen, China) until room temperature, and the weight was A_0_. Petroleum ether (5 mL) was shaken vigorously at room temperature for 5 min with FS, FG, SS, and SG (0.5 g). The solvent was recovered by filtration and the residue was washed with petroleum ether (5 mL). The surface oil was calculated according to the mass difference of the pre-weight glass beaker (A_2_).

The extractor was treated the same way as the glass beaker and weighed as A_1_: by precisely weighing FS, FG, SS, and SG (0.5 g), and extracted with petroleum ether at 80℃ in a Soxhlet apparatus (Hanon, SOX406, Shanghai, China) for 4 h. After extraction, the solvent was evaporated completely to constant weight. The total oil was calculated according to the mass difference of the pre-weight extractor (A3) [18]:

EE (%) = 1 − (Surface oil/Total oil) × 100%(1)

Surface oil = A_2_ − A_0_, Total oil = A_3_ − A_1_

#### 2.3.3. Scanning Electron Microscopy (SEM) Analysis

A JSM-6610 LV microscope (JEOL, Tokyo, Japan) was used to observe the FS, FG, SS, and SG microstructures. Samples were attached to the table with conductive double-sided tape and gold sprayed on the surface (Bal-Tec Sputter Coater SCD 050, New York, NY, USA). The SEM was performed at 10 kV with a working distance of 9.5–9.7 mm and magnifications of 800–900×.

#### 2.3.4. Differential Scanning Calorimetry (DSC) Analysis

To verify the effectiveness of the encapsulation procedure, DSC (Perkin Elmer, STA8000, Waltham, MA, USA) analysis was performed for PPO, SPI, GA, MD, FS, SS, FG, and SG. The sample was weighed into a 150 μL closed alumina crucible and its thermal behavior was analyzed in the temperature range 25–600 °C. The scans were performed under nitrogen, at a ramp rate of 10 °C/min.

#### 2.3.5. Fourier Transform Infrared (FTIR) Spectroscopic Analysis

The FTIR spectra of SPI, GA, MD, FS, SS, FG, and SG were recorded on an iS50 FTIR (Nicolet, Madison, WI, USA) at room temperature, in the wavenumber range of 500–4000 cm^−1^ with a resolution of 4 cm^−1^.

### 2.4. Volatile Compound Analysis

#### 2.4.1. Head Space-Solid Phase Microextraction/Gas Chromatography–Mass Spectroscopy (HS-SPME/GC-MS) Analysis

The above results showed that the EE, morphology, and thermal stability of SS and SG were superior to FS and FG under the same formula; therefore, SG and SS were selected for the flavor retention test. The method is outlined below.

PPO and the different microcapsule preparations were exposed to air for 15 days and analyzed every 5 days. The PPO sample was diluted to the same oil concentration as in the emulsion formulation used to make the microcapsules, then the sample (0.25 g) was mixed with distilled water (2.5 g) and 10 μL of 2-Octanol (0.25 mg/mL, methanol) as an internal standard in a 15 mL headspace bottle. The bottle was sealed and maintained at 80 °C in a water bath. An SPME fiber extracted with divinylbenzene-carboxen-polydimethylsiloxane (50/30 μm DVB/CAR/PDMS, Supelco, Inc., Bellefonte, USA) was inserted into the headspace section of the sample bottle, heated for 20 min, then removed and inserted into the GC-MS (Perkin Elmer, Waltham, USA) injection port for analysis. Desorption was performed at 250 °C for 7 min. The volatiles collected by the SPME fiber were analyzed by GC-MS, as described previously [25]. An HP-5MS quartz capillary column (30 m × 0.250 mm × 0.25 μm) was used, with an initial temperature of 40 °C for 2 min, increased at 6 °C/min to 220 °C, then at 20 °C/min to 280 °C, and held for 10 min. The carrier gas was high-purity helium (99.999%), the flow rate was 1 mL/min, the pre-column pressure was 69.8 kPa, and the split ratio was 20:1. The mass spectrometer was operated in electron impact mode (70 eV) and masses were scanned over a range of 30–330 *m*/*z*. The temperatures of the mass spectrum transmission line and ion source were 250 °C and 230 °C, respectively. A retention index (RI) was calculated for each volatile compound using the retention times of a homologous series of C_7_–C_30_ n-alkanes. Mass spectral comparisons were based on the NIST 14 mass spectral database. The relative concentrations of the flavor substances were semi-quantified and calculated according to the content and peak area of 2-Octanol, as follows [25]:

Flavor concentration = [(A_i_ × A_j_)/A_0_] × 100% (2)

where A_i_ is the concentration of 2-Octanol, A_j_ is the peak area of the flavor substance to be measured, and A_0_ is the peak area of 2-Octanol.

#### 2.4.2. Principal Component Analysis (PCA)

GC-MS results were expressed as mean ± standard deviation (SD). The data were subjected to PCA (41 volatile compounds and 1476 variables), using SIMCA 14.1 (Umetrics, Malmo, Sweden).

#### 2.4.3. Calculation of the Odor Activity Value (OAV)

The contribution of each compound to the overall PPO aroma depends on its concentration and odor threshold. In general, compounds with OAVs ≥ 1 are considered key aroma compounds [26]. In this research, the OAV was calculated as the ratio of the concentration of the compound to its odor threshold in water [27]. Thresholds were obtained from the literature [28].

### 2.5. Determination of Antioxidant Capacity

The PPO and microcapsules were stored at ambient temperature for 15 days, during which the following 3 methods were used to assess antioxidant capacity at intervals of 5 days, followed by calculation of the free radical scavenging rate.

#### 2.5.1. DPPH Clearance Rate

The DPPH clearance rate was determined, as described previously [29]. Microcapsules (200 mg) were ultrasonically (400 W) mixed with deionized water (10 mL) at room temperature for 1 min, then centrifuged at 1611 g for 10 min. For each sample, supernatant (2 mL) was mixed with DPPH (2 mL, 1 mmol/L, in anhydrous ethanol), with the same volume of water as the control, and incubated at room temperature for 30 min, protected from light. Finally, the absorbance at 517 nm was measured with a UV-VIS spectrophotometer, Genesys 10S (Thermo Scientific, Waltham, MA, USA), and ethanol as the blank. The above experiments were repeated, with VC serving as a positive control, and the DPPH free radical scavenging activity (SA_DPPH_) was calculated as follows:

SA_DPPH_ (%) = 1 − (A_sample_ − A_blank_)/A_control_ × 100% (3)

where A_sample_ is the absorbance of the sample solution, A_control_ is the absorbance of the deionized water control, and A_blank_ is the absorbance of the absolute ethanol blank.

#### 2.5.2. Hydroxyl Radical Scavenging

Two centrifuge tubes of sample (2 mL) and one of distilled water (2 mL) were treated with FeSO_4_ (2 mL, 6 mmol/L), then H_2_O_2_ (2 mL, 6 mmol/L). After thorough mixing, the mixtures were left to stand for 10 min, then treated with salicylic acid (2 mL, 6 mmol/L), distilled water (2 mL), and a second aliquot of salicylic acid. After thorough mixing, the tubes were left to stand for 30 min. The absorbance at 510 nm was measured against a distilled water blank. The aforementioned experiments were repeated, with VC serving as a positive control, and the hydroxyl radical scavenging rate was calculated as follows:

Hydroxyl radical scavenging rate (%) = [1 − (A_i_ − A_j_)/A_0_] × 100% (4)

where A_i_ and A_j_ are the absorbances of the duplicate samples and A_0_ is the absorbance of the water blank.

#### 2.5.3. Clearance of Lipid Hydroperoxides

Hydroperoxide clearance was determined, as described previously, with minor modifications [30]. Thiobarbituric acid-reactive substances (TBARS) were prepared by dissolving trichloroacetic acid (TCA, 15 g), thiobarbituric acid (TBA, 0.375 g), and HCl (2.1 mL) in distilled water (100 mL). The liposomal PBS dispersion system (LLS) was prepared by adding lecithin (300 mg) to PBS (30 mL, pH 7.4). Sample (or water for the control, 1.0 mL), LLS (1.0 mL), FeCl_3_ (1.0 mL, 0.4 mmol/L), and ascorbic acid (1.0 mL, 0.4 mmol/L) were mixed by vortexing, then maintained at 37 °C for 60 min in the dark. TBARS reagent (2.0 mL) was added, and the mixture heated at 95 °C for 15 min, cooled, then centrifugated at 1611 g for 10 min. The absorbance of the samples and the control was measured at 532 nm, against a water blank. The aforementioned experiments were repeated, with VC serving as a positive control, and the lipid hydroperoxide inhibition rate was calculated as follows:

Lipid hydroperoxide inhibition rate (%) = (1 − A/A_0_) × 100% (5)

where A is the sample absorbance and A_0_ is that of the water blank.

### 2.6. Statistical Analysis

All measurements were repeated three times. SPSS 27 software (SPSS Inc., Chicago, IL, USA) was used to calculate one-way analysis of variance (ANOVA), and Duncan’s new multiple range test was used for comparisons among samples, with *p* < 0.05 considered significantly different. Origin 2021 (OriginLab, Northampton, MA, USA) and SIMICA 14.1 software (Umetrics, Umea, Sweden) were used for data presentation.

## 3. Results

### 3.1. Characteristic Analysis of POMs

#### 3.1.1. Particle Size and EE

The particle size significantly influences the flow characteristics and dispersion properties of microcapsule powders. At present, there are studies using sodium octenyl succinate starch (OSA starch) and tea polyphenols (TPs) in spray-drying to encapsulate PPO, and the EE is controlled at 88.13 ± 1.48% [31]. In this paper, the particle size, surface oil content, and EE of POMs, prepared using different drying methods and encapsulating agents, were significantly improved compared with previous studies. The different preparation methods and materials had a significant influence on these parameters (*p* < 0.05). The particle sizes of different microcapsules exhibited unimodal distributions, providing evidence for the uniformity of particle size. Additionally, spray-dried microcapsules demonstrated smaller particle sizes and lower surface oil contents, indicating more effective control over particle size and homogeneity. Moreover, they exhibited superior embedding effects for PPO compared to freeze-dried microcapsules, resulting in a higher encapsulation efficiency (Table 1).

The EE appears to be related to the microcapsule drying method, the encapsulating agent, and the formulation of the microcapsules. The EE of spray-dried GA/MD microcapsules (SG) was 4.24% higher than that of spray-dried SPI and MD microcapsules (SS), apparently because of the higher viscosity of GA. The particle size of SG was 0.03 μm smaller than that of SS, which may be due to the molecular weight of the raw material itself and the high temperature during spray-drying, causing the microcapsule surface depression [32].

#### 3.1.2. SEM Analysis

**Figure 2:**
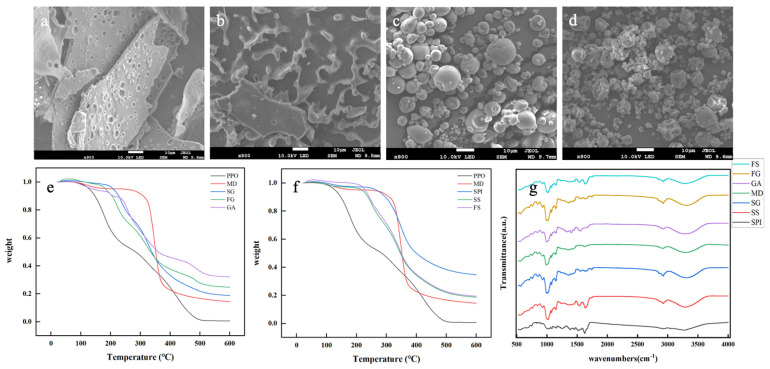
Physicochemical properties of the microcapsules observed by SEM, DSC, and FTIR: (**a**–**d**) SEM observed the microstructures of FG, FS, SG, and SS. (**e**,**f**) DSC tested the thermal stability of the FG, SG, FS, SS, and raw materials. (**g**) The structural stability of the four microcapsules and their raw materials was determined by FTIR.

The morphology of microcapsules prepared with different encapsulating agents was determined by SEM (Figure 2). The freeze-dried microcapsules (Figure 2a,b) were flaky, sharp-edged, amorphous, and porous. These structures were formed by freezing of water and sublimation of ice during drying. In contrast, the spray-dried microcapsules had a spherical morphology, and the GA-based microcapsules (Figure 2c) had smooth surfaces, whereas those based on SPI (Figure 2d) had wrinkled surfaces and numerous surface depressions. The irregularity of the SS surface can be attributed to protein denaturation or rapid water evaporation resulting from the high inlet temperature of the spray-dryer [33].

#### 3.1.3. DSC Analysis

Thermal degradation plots of the microcapsules were determined by DSC (Figure 2e,f). PPO degradation started at 100 °C, then proceeded in a relatively linear manner until complete carbonization at 500 °C. This behavior resulted from a combination of the different thermal stabilities of the various components of PPO. In contrast, the microcapsules were essentially stable up to 200 °C, then decomposed more rapidly than PPO, to reach complete carbonization at 500 °C. The temperature-dependent mass loss from the microcapsules occurred in three main stages. During the initial stage (20–200 °C), the gradual mass loss corresponded to water evaporation and the release of volatile substances. In the second stage (200–400 °C), all the plots had a sharp drop, resulting from rupture of the capsule wall, oil leakage, and thermal decomposition of the oil and other components. In the third stage (400–550 °C), the mass loss was slow and corresponded to complete carbonization of residual proteins and polysaccharides and the completion of thermal decomposition. The thermal stability of SG was slightly higher than that of FG (Figure 2e), while that of FS and SS was similar (Figure 2f) but slightly higher than that of FG and SG. Most importantly, the stability of the PPO microcapsules was markedly higher than that of PPO, in agreement with a previous report [3].

#### 3.1.4. FTIR Analysis

FTIR analysis provides information on the molecular structures and chemical bonds in molecules of any size. All samples (except SPI) had wide absorption peaks at 3000–3500 cm^−1^, corresponding to the hydroxyl group of polysaccharides (Figure 2g) [34]. PPO had a strong tensile vibration peak corresponding to –HC=CH– at 3100~2700 cm^−1^, indicating the presence of unsaturated fatty acid [18], which was also present in the spectra of all four microcapsule types, indicating effective encapsulation of the oil. GA, MD, and SPI had weak absorption peaks at 2930 cm^−1^, characteristic of C–H bonds [34]. SPI, SS, and FS had strong characteristic peaks associated with C=O bonds around 1730 cm^−1^ [18], and all samples had strong peaks at 1000 cm^−1^, characteristic of C–C bonds [35]. The absence of any new absorption peak in the microcapsule spectra indicated that the chemical structures of PPO and the encapsulating agents remained unchanged during microcapsule preparation.

### 3.2. GC-MS Analysis

#### 3.2.1. Composition and Content of Volatile Compounds

**Figure 3:**
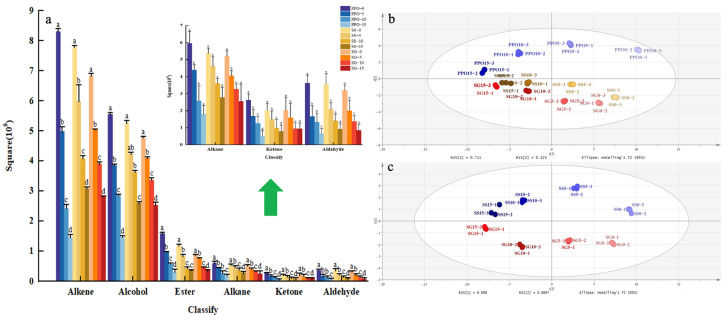
Change in flavor substances in PPO, SS, and SG during the 15-day storage period: (**a**) Trends of volatile compound concentrations in PPO, SS, and SG over 15 days. (**b**,**c**) PCA results.

The compound classes and content of all flavor substances in PPO and the microcapsules after storage at 25 °C for 0, 5, 10, and 15 days were determined by HS-SPME/GC-MS (Figure 3a). The concentrations of all detected compounds in PPO, SS, and SG decreased markedly with the increased air-exposure time (*p* < 0.05), but the decreases in SS and SG were much smaller than those in PPO. The main flavors were from six compound classes: aldehydes, alcohols, ketones, olefins, alkanes, and esters. After 15 days, a large number of alkenes and alcohol in PPO evaporated or degraded, and the loss rate was significantly higher than that in SS and SG. Encapsulation, especially with SS, markedly inhibited the loss of flavor in PPO during air exposure (Figure 3a). Overall, half of the volatile compounds in SG were lost after 15 days, significantly lower than that for PPO and SS (*p* < 0.05), thereby demonstrating a useful flavor retention effect (Figure S1).

#### 3.2.2. Principal Component Analysis (PCA)

PCA is a clustering statistical analysis method. Interrelated original variables are transformed into several orthogonal principal component variables through orthogonal transformation, then differences between samples are determined from the relative contributions of the principal component variables [36]. PCA models were constructed to determine the variability of the 41 volatile flavors identified in PPO, SS, and SG samples, prepared by spray-drying at different air-exposure times (Figure 3b). The PCA model parameters were correlation coefficient (R2) = 0.874 and predictive ability (Q) = 0.807, indicating high data reliability. Notably, significant differences were observed among the three sample types (*p* < 0.05). The microcapsule samples with different air-exposure times were distributed across the 3rd and 4th quadrants, whereas PPO was predominantly distributed across the 1st and 2nd quadrants (Figure 3b), indicating a significant flavor difference between PPO and the microcapsules during air exposure. The larger separation between SG than SS sample points and PPO indicated a greater flavor content difference in SG.

PCA was also used to visualize the differences between SG and SS with air-exposure time, yielding R2 = 0.96 and Q = 0.888, indicating very good discrimination between samples (Figure 3c). SS samples stored for 0, 5, 10, and 15 days were distributed across quadrants 1 and 2, whereas SG samples were distributed across quadrants 3 and 4. This indicated significant differences in both the flavor composition and content of SG and SS.

#### 3.2.3. Volatile Flavor Compound Analysis

The concentrations of the 41 identified flavors in PPO are shown in Table 2. Encapsulation had a significant effect on the decrease in concentration of the compounds resulting from degradation and evaporation (*p* < 0.05). The most abundant compound in PPO at day 0 was linalool, followed by D-limonene, sabinene, β-myrcene, and linalyl acetate. These findings are consistent with previous reports [3,37]. After 15 days, the concentrations of these aroma components decreased, which may be attributed to the high volatility of these compounds or their susceptibility to degradation, oxidation, and isomerization [38].

**Figure 4:**
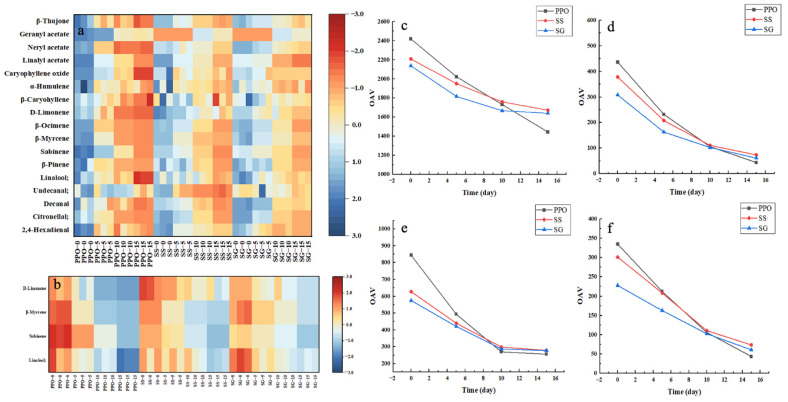
Changes in the OAV of flavor substances during the storage period: (**a**) Heat map of substances with OAV > 1 for 15 days. (**b**) Heat map of core flavor substances’ OAV changes over 15 days. (**c**–**f**) Changes in OAV of linalool, sabinene, β-myrcene, and D-limonene in PPO, SS, and SG during the storage period.

The odor activity value (OAV) of a flavor indicates its contribution to the overall flavor and is related to its concentration and odor detection threshold [39]. Of the 41 identified compounds, there were 17 with OAVs ≥ 1 (Table 3), comprising 4 aldehydes, 1 alcohol, 8 olefins, 3 esters, and 1 ketone (Figure 4a). Of these, four were established as key flavors, with OAVs > 100, namely, linalool, β-myrcene, sabinene, and D-limonene. Heat map analysis showed that the OAVs of these compounds changed significantly during air exposure for 15 days (Figure 4b). These compounds are also significant flavors in other essential oils and exhibit distinct flavor characteristics. Linalool is abundant in lavender [40], sweet orange [7], cinnamon [5], and other essential oils. It exhibits a fresh floral and woody aroma [41]. Olefin substances primarily possess a refreshing grassy scent and pungent taste [41]. β-Myrcene, a common food flavoring agent and food additive, can be found in the essential oils of various plants, such as hops, hemp, lemongrass, verbena, and laurel [6]. D-Limonene is present in most citrus fruit essential oils and has a citrus scent [42]. The essential oil of Lepechinia betonicifolia has a high content of sabinene (27.98%), which contributes notably to the aromatic profiles associated with “citrus” and “minty” scents [36]. In PPO, the compounds linalool and β-myrcene contribute to the spicy, woody, and green aroma. The citrus scent is derived from D-limonene. α-Pinene, sabinene, and γ-terpinene provide a pine wood flavor to PPO [43]. Combinations of these compounds may exhibit additive, synergistic, or antagonistic interactions that influence the perception of PPO’s aroma.

The variations in OAV of the four key flavors were determined during the 15-day air-exposure period (Figure 4b). There was a significant decrease in the OAVs of all four compounds during air exposure. On day 10, the OAV of linalool in SS exceeded PPO. Within 15 days, the loss rate of linalool in POMs was half that of PPO (Figure 4e). There was no significant difference in the loss rate of β-myrcene between SS and SG (Figure 4e), but the loss rates of sabinene and D-limonene in SG were lower than that of SS (Figure 4d,f). In short, at day 15, OAV values of the microcapsule samples were significantly higher than PPO values. Encapsulation appeared to result in a considerably reduced evaporation of the main flavor, thereby functioning as a flavor retention formulation of PPO. SG had the best flavor retention effect.

### 3.3. Antioxidant Capacity

Some researchers found that D-limonene has the effects of antioxidation, inhibition of lipid peroxidation, and resistance to cell damage induced by free radicals [42]. Ciftci et al. added β-myrcene to corn oil (200 mg/kg/d) and administered it to Sprague–Dawley (SD) rats via gastric irrigation. After 60 days, the TBARS content in liver tissue decreased by 21 ± 0.5 nmol/g, which proved that β-myrcene has a positive antioxidant ability [44]. It was observed that sabinene possesses inhibitory activity against acetylcholinesterase [36]. The above studies indicate that these flavors not only confer favorable flavor to PPO but also exhibit antioxidant and other biological activities.

**Figure 5:**
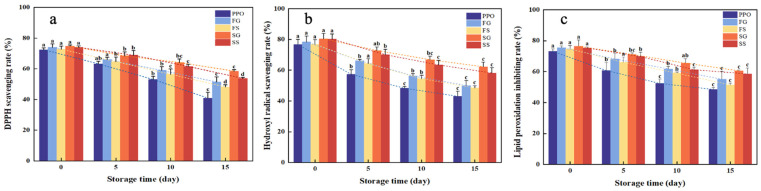
Antioxidant analysis of PPO, SS, SG, FS, and FG over 15 days: (**a**) DPPH clearance rate, (**b**) scavenging hydroxyl radicals, and (**c**) lipid hydroperoxide clearance.

The antioxidant analysis results of PPO and microcapsules with different storage times consistently demonstrated that the incorporation of embedded microcapsules effectively reserved the antioxidant capacity of PPO (Figure 5). All four encapsulation methods increased the three antioxidant capacity measures all throughout the air-exposure period (*p* < 0.05). Encapsulation enhanced the antioxidant capacity of PPO, probably because of the high retention effect of microcapsules on flavor substances. The shell structure of the microcapsules efficiently encapsulated the active ingredient in oil, decreasing its exposure time to oxygen and thereby augmenting its antioxidant properties.

The DPPH clearance, hydroxyl radical clearance, and lipid hydroperoxide clearance of PPO, SG, and SS showed similar magnitudes due to the antioxidant activity of PPO itself on day 0 (Figure 5), then decreased with time. The DPPH clearance decreased linearly due to the rapid oxidation of PPO over 15 days (Figure 5a). The scavenging rate of the hydroxyl radical and the lipid hydroperoxide clearance rates decreased exponentially with time (Figure 5b,c). There were significant differences in antioxidant capacity between PPO and microcapsules (*p* < 0.05).

In general, the relatively porous structure of the freeze-dried surface permitted faster evaporation of volatile PPO components, thus reducing the EE, which resulted in a higher antioxidant capacity of spray-dried microcapsules than freeze-dried microcapsules. SG had a higher antioxidant capacity than SS, which agrees with a previous report that large particle-size spray-dried powders had poor oxidation stability compared to small particle-size powders [45].

## 4. Conclusions

Soy protein isolate (SPI) or gum Arabic (GA), combined with maltodextrin, were used as encapsulating agents for the encapsulation of Prickly ash peel oleoresin (PPO) by spray-drying or freeze-drying. The physicochemical properties of the four encapsulated preparations were compared with those of PPO. All microcapsules had excellent dispersibility in water, which effectively changed the physical properties of PPO and solved its low water solubility. Spray-dried microcapsules had a smaller particle size, reduced surface oil content, enhanced water-binding capacity, and superior antioxidant capacity, compared with freeze-dried microcapsules. Furthermore, microcapsules with GA as the encapsulating agent had higher thermal stability and oxidation resistance than those with SPI.

The analysis of PPO and the microcapsules using HS-SPME-GC-MS identified 41 flavors, and linalool, sabinene, β-myrcene, and D-limonene were, quantitatively, the major flavor components. The concentrations of these flavors decreased during air exposure, with SG having the highest retention of these compounds. The DPPH scavenging, hydroxyl radical scavenging, and lipid peroxide scavenging capabilities of SG were higher than those of the other encapsulation methods, and all were markedly higher than those of PPO. These findings demonstrated that the microcapsules effectively preserved the flavors of PPO for a longer time and possessed excellent antioxidant properties. Consequently, these results are anticipated to facilitate the development of novel Zanthoxylum products as food ingredients.

## Figures and Tables

**Table 1:** Recipe, oil content, encapsulation rate, and size distribution of the microcapsules.

Name	Method	Wall Material	Surface Oil Content	EE	Size (μm)
FS	Freeze-drying	SPI, MD	2.50 ± 0.42% ^a^	86.60 ± 0.38% ^a^	3.26 ± 0.09 ^c^
SS	Spray-drying	SPI, MD	1.89 ± 0.20% ^b^	88.07 ± 0.26% ^a^	2.42 ± 0.08 ^a^
FG	Freeze-drying	GA, MD	2.48 ± 0.33% ^a^	87.71 ± 0.10% ^a^	2.94 ± 0.12 ^b^
SG	Spray-drying	GA, MD	0.60 ± 0.11% ^c^	92.31 ± 0.31% ^b^	2.39 ± 0.15 ^a^

SS (SPI and MD) and SG (GA and MD) are spray-dried microcapsules, and the corresponding freeze-dried microcapsules are FS and FG, respectively. Values represent the means ± standard deviation; *n* = 3. Different letters (^a^, ^b^, ^c^) in each column indicate significant differences (*p* < 0.05).

**Table 2:** The concentrations of each compound by GC-MS analysis in PPO, SS, and SG over 15 days.

Number	Name	Chemical Formula	CAS Number	Concentration (μg/g)											
				Day 0			Day 5			Day 10			Day 15		
				PPO	SS	SG	PPO	SS	SG	PPO	SS	SG	PPO	SS	SG
1	Linalool	C_10_H_18_O	78-70-6	14.51 ± 3.40 ^a^	13.26 ± 0.83 ^a^	12.82 ± 2.38 ^a^	12.13 ± 1.96 ^a^	10.09 ± 0.70 ^a^	9.70 ± 1.00 ^b^	9.91 ± 1.21 ^ab^	10.09 ± 0.70 ^ab^	7.29 ± 0.20 ^bc^	7.75 ± 0.95 ^b^	8.47 ± 0.23 ^b^	8.64 ± 0.86 ^c^
2	D-Limonene	C_10_H_16_	5989-27-5	11.38 ± 1.68 ^a^	11.26 ± 0.52 ^a^	8.73 ± 1.81 ^a^	7.21 ± 1.75 ^a^	7.08 ± 1.06 ^b^	5.54 ± 0.73 ^ab^	3.60 ± 0.24 ^ab^	3.77 ± 1.25 ^c^	3.50 ± 0.82 ^ab^	1.49 ± 0.05 ^b^	2.51 ± 1.88 ^d^	2.08 ± 0.29 ^b^
3	Sabinene	C_10_H_16_	3387-41-5	6.99 ± 1.45 ^a^	4.29 ± 0.34 ^a^	3.25 ± 0.24 ^a^	3.71 ± 0.82 ^b^	3.33 ± 0.03 ^b^	2.61 ± 0.15 ^b^	1.69 ± 0.47 ^c^	1.77 ± 0.10 ^c^	1.64 ± 0.06 ^bc^	0.70 ± 0.07 ^c^	1.18 ± 0.06 ^c^	0.98 ± 0.23 ^c^
4	Linalyl acetate	C_12_H_20_O_2_	115-95-7	4.96 ± 0.92 ^a^	3.80 ± 0.30 ^a^	3.24 ± 0.23 ^a^	3.00 ± 0.45 ^b^	2.56 ± 0.06 ^b^	2.41 ± 0.33 ^b^	2.13 ± 0.28 ^c^	2.09 ± 0.09 ^c^	1.74 ± 0.43 ^bc^	1.24 ± 0.10 ^c^	1.34 ± 0.23 ^d^	1.01 ± 0.29 ^c^
5	β-Myrcene	C_10_H_16_	123-35-3	4.13 ± 0.78 ^a^	3.07 ± 0.23 ^a^	2.82 ± 0.15 ^a^	2.42 ± 0.36 ^b^	2.16 ± 0.03 ^b^	2.06 ± 0.11 ^b^	1.32 ± 0.17 ^c^	1.46 ± 0.07 ^c^	1.66 ± 0.05 ^c^	1.25 ± 0.09 ^c^	1.37 ± 0.23 ^d^	1.36 ± 0.21 ^d^
6	β-Copaene	C_15_H_24_	18252-44-3	1.96 ± 0.25 ^a^	1.32 ± 0.18 ^a^	1.10 ± 0.06 ^a^	1.28 ± 0.16 ^ab^	1.03 ± 0.15 ^a^	0.87 ± 0.13 ^b^	1.17 ± 0.14 ^ab^	0.91 ± 0.21 ^ab^	0.77 ± 0.12 ^b^	0.88 ± 0.24 ^b^	0.75 ± 0.06 ^b^	0.70 ± 0.10 ^b^
7	α-Terpineol	C_10_H_18_O	98-55-5	1.73 ± 0.17 ^a^	1.36 ± 0.26 ^a^	1.02 ± 0.09 ^a^	1.37 ± 0.33 ^a^	1.18 ± 0.12 ^a^	0.87 ± 0.08 ^a^	0.62 ± 0.19 ^b^	0.76 ± 0.07 ^b^	0.70 ± 0.05 ^b^	0.27 ± 0.03 ^b^	0.36 ± 0.09 ^c^	0.31 ± 0.05 ^c^
8	β-Caryophyllene	C_15_H_24_	87-44-5	1.53 ± 0.24 ^a^	1.23 ± 0.13 ^a^	1.08 ± 0.07 ^a^	1.25 ± 0.15 ^b^	1.29 ± 0.06 ^a^	1.07 ± 0.13 ^b^	1.17 ± 0.14 ^b^	1.11 ± 0.07 ^b^	1.01 ± 0.06 ^c^	1.09 ± 0.16 ^b^	0.95 ± 0.08 ^b^	1.00 ± 0.10 ^c^
9	* Spathulenol *	C_15_H_24_O	6750-60-3	1.28 ± 0.23 ^a^	0.93 ± 0.10 ^a^	0.86 ± 0.05 ^a^	1.11 ± 0.16 ^a^	0.72 ± 0.01 ^b^	0.64 ± 0.15 ^b^	0.10 ± 0.01 ^b^	0.42 ± 0.02 ^b^	0.42 ± 0.01 ^c^	0.09 ± 0.01 ^b^	0.12 ± 0.01 ^c^	0.09 ± 0.01 ^d^
10	* Caryophyleneoxide *	C_15_H_24_O	1139-30-6	1.28 ± 0.37 ^a^	0.95 ± 0.24 ^a^	0.65 ± 0.07 ^a^	0.69 ± 0.10 ^b^	0.62 ± 0.04 ^b^	0.59 ± 0.05 ^b^	0.65 ± 0.08 ^bc^	0.57 ± 0.02 ^b^	0.46 ± 0.02 ^c^	0.61 ± 0.14 ^c^	0.47 ± 0.03 ^b^	0.44 ± 0.13 ^d^
11	α-Pinene	C_10_H_16_	80-56-8	1.09 ± 0.24 ^a^	0.78 ± 0.04 ^a^	0.68 ± 0.06 ^a^	0.64 ± 0.08 ^b^	0.61 ± 0.13 ^a^	0.57 ± 0.07 ^b^	0.30 ± 0.07 ^c^	0.32 ± 0.04 ^b^	0.30 ± 0.04 ^c^	0.13 ± 0.08 ^c^	0.16 ± 0.03 ^c^	0.17 ± 0.05 ^d^
12	* β-Ocimene *	C_10_H_16_	13877-91-3	0.90 ± 0.25 ^a^	0.70 ± 0.07 ^a^	0.63 ± 0.06 ^a^	0.40 ± 0.07 ^b^	0.52 ± 0.01 ^b^	0.51 ± 0.05 ^b^	0.22 ± 0.04 ^b^	0.29 ± 0.01 ^c^	0.30 ± 0.01 ^c^	0.13 ± 0.01 ^b^	0.17 ± 0.04 ^d^	0.16 ± 0.04 ^c^
13	* 2,4-Hexadienal *	C_6_H_8_O	142-83-6	0.86 ± 0.14 ^a^	0.65 ± 0.07 ^a^	0.47 ± 0.07 ^a^	0.40 ± 0.10 ^b^	0.37 ± 0.01 ^b^	0.23 ± 0.06 ^b^	0.21 ± 0.12 ^bc^	0.30 ± 0.02 ^b^	0.16 ± 0.03 ^b^	0.12 ± 0.06 ^c^	0.16 ± 0.01 ^c^	0.13 ± 0.03 ^b^
14	* α-Ocimene *	C_10_H_16_	502-99-8	0.76 ± 0.18 ^a^	0.79 ± 0.18 ^a^	0.37 ± 0.02 ^a^	0.55 ± 0.14 ^b^	0.46 ± 0.03 ^b^	0.20 ± 0.11 ^b^	0.16 ± 0.05 ^c^	0.30 ± 0.02 ^c^	0.17 ± 0.15 ^c^	0.10 ± 0.03 ^c^	0.23 ± 0.01 ^c^	0.14 ± 0.01 ^c^
15	* Terpinen-4-ol *	C_10_H_18_O	20126-76-5	0.71 ± 0.13 ^a^	0.60 ± 0.06 ^a^	0.51 ± 0.06 ^a^	0.56 ± 0.09 ^ab^	0.46 ± 0.07 ^b^	0.37 ± 0.01 ^b^	0.42 ± 0.03 ^bc^	0.33 ± 0.01 ^c^	0.26 ± 0.02 ^c^	0.25 ± 0.03 ^c^	0.25 ± 0.04 ^c^	0.18 ± 0.01 ^d^
16	* α-Humulene *	C_15_H_24_	6753-98-6	0.70 ± 0.13 ^a^	0.95 ± 0.24 ^a^	0.55 ± 0.07 ^a^	0.46 ± 0.06 ^b^	0.62 ± 0.04 ^a^	0.32 ± 0.04 ^b^	0.35 ± 0.11 ^b^	0.42 ± 0.02 ^b^	0.30 ± 0.02 ^bc^	0.25 ± 0.03 ^b^	0.34 ± 0.04 ^bc^	0.27 ± 0.03 ^c^
17	* 2-Methylhexadecane *	C_17_H_36_	1560-92-5	0.68 ± 0.12 ^a^	0.57 ± 0.04 ^a^	0.45 ± 0.02 ^a^	0.61 ± 0.09 ^a^	0.53 ± 0.01 ^b^	0.43 ± 0.02 ^a^	0.33 ± 0.04 ^b^	0.44 ± 0.02 ^bc^	0.38 ± 0.02 ^b^	0.21 ± 0.02 ^b^	0.32 ± 0.02 ^c^	0.30 ± 0.03 ^b^
18	* β-Thujone *	C_10_H_16_O	471-15-8	0.66 ± 0.11 ^a^	0.48 ± 0.04 ^a^	0.41 ± 0.04 ^a^	0.30 ± 0.08 ^b^	0.36 ± 0.01 ^b^	0.36 ± 0.02 ^a^	0.24 ± 0.06 ^b^	0.24 ± 0.01 ^c^	0.29 ± 0.02 ^b^	0.14 ± 0.03 ^b^	0.18 ± 0.01 ^d^	0.22 ± 0.01 ^c^
19	2-Methyltetradecane	C_15_H_32_	1560-95-8	0.65 ± 0.07 ^a^	0.45 ± 0.03 ^a^	0.41 ± 0.04 ^a^	0.35 ± 0.05 ^b^	0.34 ± 0.01 ^b^	0.36 ± 0.02 ^ab^	0.22 ± 0.03 ^bc^	0.30 ± 0.01 ^c^	0.31 ± 0.02 ^b^	0.18 ± 0.01 ^c^	0.26 ± 0.02 ^c^	0.24 ± 0.03 ^c^
20	β-Elemene	C_15_H_24_	515-13-9	0.09 ± 0.02 ^a^	0.07 ± 0.00 ^a^	0.06 ± 0.00 ^a^	0.08 ± 0.01 ^a^	0.06 ± 0.00 ^ab^	0.06 ± 0.01 ^a^	0.07 ± 0.00 ^a^	0.05 ± 0.01 ^bc^	0.05 ± 0.00 ^a^	0.05 ± 0.00 ^a^	0.05 ± 0.01 ^c^	0.05 ± 0.00 ^a^
21	Phenethyl acetate	C_10_H_12_O_2_	103-45-7	0.05 ± 0.01 ^a^	0.03 ± 0.01 ^a^	0.05 ± 0.01 ^a^	0.04 ± 0.01 ^b^	0.03 ± 0.01 ^b^	0.04 ± 0.00 ^b^	0.01 ± 0.00 ^b^	0.02 ± 0.00 ^c^	0.03 ± 0.01 ^c^	0.00 ± 0.00 ^b^	0.01 ± 0.00 ^d^	0.01 ± 0.00 ^d^
22	Tetradecane	C_14_H_30_	629-59-4	0.52 ± 0.09 ^a^	0.40 ± 0.01 ^a^	0.33 ± 0.02 ^a^	0.34 ± 0.05 ^b^	0.29 ± 0.03 ^b^	0.31 ± 0.02 ^b^	0.19 ± 0.03 ^c^	0.22 ± 0.03 ^b^	0.25 ± 0.01 ^b^	0.17 ± 0.01 ^c^	0.18 ± 0.03 ^c^	0.16 ± 0.01 ^c^
23	Nerolidol	C_15_H_26_O	40716-66-3	0.49 ± 0.09 ^a^	0.36 ± 0.03 ^a^	0.25 ± 0.01 ^a^	0.28 ± 0.04 ^b^	0.18 ± 0.00 ^b^	0.16 ± 0.01 ^b^	0.16 ± 0.02 ^b^	0.14 ± 0.01 ^b^	0.13 ± 0.00 ^bc^	0.16 ± 0.01 ^b^	0.13 ± 0.01 ^c^	0.12 ± 0.01 ^c^
24	γ-Terpinene	C_10_H_16_	99-85-4	0.49 ± 0.09 ^a^	0.36 ± 0.03 ^a^	0.25 ± 0.01 ^a^	0.28 ± 0.04 ^b^	0.18 ± 0.00 ^b^	0.16 ± 0.01 ^b^	0.16 ± 0.02 ^bc^	0.14 ± 0.01 ^c^	0.13 ± 0.00 ^b^	0.16 ± 0.01 ^c^	0.13 ± 0.01 ^d^	0.12 ± 0.01 ^c^
25	8-Hetadecene	C_17_H_34_	2579-04-6	0.44 ± 0.08 ^a^	0.00 ± 0.00 ^a^	0.00 ± 0.00 ^a^	0.32 ± 0.05 ^b^	0.36 ± 0.01 ^b^	0.00 ± 0.00 ^a^	0.24 ± 0.03 ^bc^	0.29 ± 0.01 ^b^	0.00 ± 0.00 ^b^	0.16 ± 0.01 ^c^	0.15 ± 0.01 ^b^	0.00 ± 0.00 ^b^
26	2-Tridecanone	C_13_H_26_O	593-08-8	0.42 ± 0.08 ^a^	0.21 ± 0.02 ^a^	0.17 ± 0.01 ^a^	0.35 ± 0.04 ^ab^	0.16 ± 0.00 ^b^	0.12 ± 0.01 ^b^	0.27 ± 0.05 ^b^	0.08 ± 0.00 ^c^	0.09 ± 0.00 ^c^	0.08 ± 0.00 ^c^	0.07 ± 0.00 ^c^	0.08 ± 0.01 ^c^
27	* 4-Thujanol *	C_10_H_18_O	546-79-2	0.42 ± 0.08 ^a^	0.21 ± 0.02 ^a^	0.17 ± 0.01 ^a^	0.35 ± 0.04 ^ab^	0.16 ± 0.00 ^a^	0.12 ± 0.01 ^a^	0.27 ± 0.05 ^bc^	0.08 ± 0.00 ^b^	0.09 ± 0.00 ^b^	0.08 ± 0.00 ^c^	0.07 ± 0.00 ^c^	0.08 ± 0.01 ^c^
28	Citronellal	C_10_H_18_O	106-23-0	0.25 ± 0.06 ^a^	0.24 ± 0.05 ^a^	0.19 ± 0.02 ^a^	0.09 ± 0.02 ^b^	0.15 ± 0.01 ^b^	0.14 ± 0.01 ^b^	0.06 ± 0.01 ^b^	0.05 ± 0.02 ^c^	0.11 ± 0.01 ^c^	0.03 ± 0.01 ^b^	0.03 ± 0.00 ^c^	0.03 ± 0.01 ^d^
29	1,7-Octadien-3-one, 2-methyl-6-methylene	C_10_H_14_O	41702-60-7	0.23 ± 0.04 ^a^	0.15 ± 0.01 ^a^	0.12 ± 0.01 ^a^	0.08 ± 0.01 ^b^	0.07 ± 0.00 ^b^	0.08 ± 0.00 ^b^	0.03 ± 0.01 ^c^	0.04 ± 0.00 ^c^	0.05 ± 0.00 ^c^	0.03 ± 0.00 ^c^	0.03 ± 0.00 ^d^	0.03 ± 0.01 ^d^
30	Decanal	C_10_H_20_O	112-31-2	0.21 ± 0.05 ^a^	0.17 ± 0.03 ^a^	0.13 ± 0.02 ^a^	0.13 ± 0.01 ^a^	0.13 ± 0.01 ^ab^	0.12 ± 0.01 ^a^	0.12 ± 0.05 ^ab^	0.10 ± 0.02 ^b^	0.10 ± 0.00 ^ab^	0.03 ± 0.05 ^b^	0.00 ± 0.00 ^c^	0.08 ± 0.02 ^b^
31	Cyclohexane, 2-ethenyl-1,1-dimethyl-3-methylene-	C_11_H_18_	95452-08-7	0.19 ± 0.04 ^a^	0.15 ± 0.01 ^a^	0.11 ± 0.00 ^a^	0.08 ± 0.01 ^b^	0.07 ± 0.00 ^b^	0.10 ± 0.01 ^a^	0.05 ± 0.01 ^bc^	0.05 ± 0.00 ^b^	0.05 ± 0.00 ^b^	0.03 ± 0.00 ^c^	0.03 ± 0.00 ^c^	0.03 ± 0.00 ^c^
32	Undecanal	C_11_H_22_O	112-44-7	0.18 ± 0.06 ^a^	0.15 ± 0.01 ^a^	0.14 ± 0.00 ^b^	0.14 ± 0.05 ^a^	0.06 ± 0.01 ^b^	0.12 ± 0.03 ^b^	0.16 ± 0.02 ^a^	0.04 ± 0.00 ^bc^	0.08 ± 0.01 ^c^	0.09 ± 0.01 ^a^	0.03 ± 0.01 ^c^	0.05 ± 0.01 ^c^
33	γ-Elemene	C_15_H_24_	3242-08-8	0.17 ± 0.03 ^a^	0.12 ± 0.01 ^a^	0.00 ± 0.00 ^a^	0.11 ± 0.02 ^b^	0.09 ± 0.00 ^b^	0.00 ± 0.00 ^a^	0.09 ± 0.01 ^b^	0.08 ± 0.00 ^bc^	0.00 ± 0.00 ^b^	0.04 ± 0.00 ^c^	0.04 ± 0.00 ^c^	0.00 ± 0.00 ^c^
34	Geranyl acetate	C_12_H_20_O_2_	105-87-3	0.17 ± 0.03 ^a^	0.00 ± 0.00 ^a^	0.00 ± 0.00 ^a^	0.14 ± 0.02 ^b^	0.00 ± 0.00 ^ab^	0.00 ± 0.00 ^b^	0.06 ± 0.01 ^b^	0.08 ± 0.01 ^b^	0.07 ± 0.00 ^c^	0.05 ± 0.01 ^b^	0.05 ± 0.01 ^c^	0.05 ± 0.00 ^c^
35	Terpinolene	C_10_H_16_	586-62-9	0.15 ± 0.03 ^a^	0.00 ± 0.00 ^a^	0.00 ± 0.00 ^a^	0.06 ± 0.01 ^b^	0.10 ± 0.00 ^b^	0.09 ± 0.01 ^b^	0.06 ± 0.01 ^b^	0.06 ± 0.01 ^c^	0.04 ± 0.00 ^c^	0.03 ± 0.00 ^b^	0.03 ± 0.00 ^c^	0.03 ± 0.00 ^d^
36	Neryl acetate	C_12_H_20_O_2_	141-12-8	0.13 ± 0.02 ^a^	0.09 ± 0.01 ^a^	0.09 ± 0.00 ^a^	0.06 ± 0.01 ^a^	0.08 ± 0.01 ^a^	0.08 ± 0.01 ^a^	0.04 ± 0.01 ^b^	0.07 ± 0.00 ^b^	0.06 ± 0.01 ^b^	0.04 ± 0.00 ^b^	0.05 ± 0.00 ^b^	0.05 ± 0.01 ^b^
37	Dodecane, 2-methyl-	C_13_H_28_	1560-97-0	0.11 ± 0.02 ^a^	0.07 ± 0.01 ^a^	0.06 ± 0.00 ^a^	0.05 ± 0.01 ^b^	0.04 ± 0.00 ^b^	0.05 ± 0.00 ^b^	0.04 ± 0.00 ^b^	0.04 ± 0.01 ^c^	0.04 ± 0.01 ^b^	0.04 ± 0.00 ^b^	0.03 ± 0.00 ^d^	0.04 ± 0.01 ^b^
38	2-Cyclohexen-1-one,4-(1-methylethyl)-	C_9_H_14_O	500-02-7	0.09 ± 0.02 ^a^	0.07 ± 0.01 ^a^	0.05 ± 0.01 ^a^	0.06 ± 0.01 ^b^	0.05 ± 0.00 ^b^	0.03 ± 0.00 ^b^	0.02 ± 0.01 ^c^	0.03 ± 0.00 ^c^	0.02 ± 0.00 ^b^	0.02 ± 0.00 ^c^	0.02 ± 0.01 ^c^	0.02 ± 0.00 ^c^
39	Cryptomeridiol	C_15_H_28_O_2_	4666-84-6	0.08 ± 0.01 ^a^	0.06 ± 0.01 ^a^	0.05 ± 0.01 ^a^	0.05 ± 0.01 ^b^	0.04 ± 0.00 ^b^	0.05 ± 0.01 ^a^	0.00 ± 0.00 ^c^	0.04 ± 0.00 ^c^	0.04 ± 0.00 ^b^	0.00 ± 0.00 ^c^	0.02 ± 0.01 ^d^	0.02 ± 0.00 ^c^
40	(-)-Germacrene-D	C_15_H_24_	317819-80-0	0.08 ± 0.02 ^a^	0.06 ± 0.01 ^a^	0.05 ± 0.01 ^a^	0.07 ± 0.01 ^b^	0.05 ± 0.01 ^ab^	0.05 ± 0.00 ^a^	0.04 ± 0.00 ^bc^	0.04 ± 0.01 ^b^	0.04 ± 0.01 ^a^	0.03 ± 0.00 ^c^	0.03 ± 0.00 ^b^	0.03 ± 0.00 ^a^
41	* α-Elemol *	C_15_H_26_O	639-99-6	0.06 ± 0.01 ^a^	0.05 ± 0.00 ^a^	0.04 ± 0.00 ^a^	0.04 ± 0.01 ^ab^	0.04 ± 0.00 ^b^	0.03 ± 0.00 ^a^	0.04 ± 0.01 ^bc^	0.04 ± 0.00 ^c^	0.03 ± 0.00 ^b^	0.03 ± 0.00 ^c^	0.03 ± 0.00 ^d^	0.03 ± 0.00 ^c^

SS (SPI and MD) and SG (GA and MD) are the spray-dried microcapsules, respectively. Values represent the means ± standard deviation; *n* = 3. Different letters (^a^, ^b^, ^c^, ^d^) in each column indicate significant differences (*p* < 0.05).

**Table 3:** OAV of flavor substances in PPO, SS, and SG over 15 days.

Number	Name	CAS Number	Threshold Value (μg/g)	OAV											
				Day 0				Day 5		Day 10				Day 15	
				PPO	SS	SG	PPO	SS	SG	PPO	SS	SG	PPO	SS	SG
1	2,4-Hexadienal	142-83-6	0.06	14.33 ± 2.26 ^a^	10.77 ± 1.17 ^a^	7.75 ± 1.11 ^a^	6.60 ± 1.74 ^b^	6.22 ± 0.05 ^b^	3.72 ± 1.06 ^b^	3.45 ± 2.06 ^bc^	4.97 ± 0.29 ^b^	2.61 ± 0.42 ^b^	2.01 ± 0.97 ^c^	2.66 ± 0.45 ^c^	2.17 ± 0.56 ^b^
2	Citronellal	106-23-0	0.10	0.25 ± 0.06 ^a^	0.24 ± 0.05 ^a^	0.19 ± 0.02 ^a^	0.09 ± 0.02 ^b^	0.15 ± 0.01 ^b^	0.14 ± 0.01 ^b^	0.06 ± 0.01 ^b^	0.05 ± 0.02 ^c^	0.11 ± 0.01 ^c^	0.03 ± 0.00 ^b^	0.03 ± 0.00 ^c^	0.03 ± 0.00 ^d^
3	Decanal	112-31-2	0.003	70.39 ± 15.66 ^a^	56.37 ± 10.63 ^a^	43.91 ± 6.24 ^a^	44.08 ± 2.63 ^a^	44.05 ± 1.12 ^ab^	38.43 ± 4.42 ^a^	40.91 ± 15.42 ^ab^	32.08 ± 7.28 ^b^	33.21 ± 0.43 ^ab^	11.15 ± 15.77 ^b^	0.00 ± 0.00 ^c^	26.27 ± 6.08 ^b^
4	Undecanal	112-44-7	0.0125	14.78 ± 5.03 ^a^	12.31 ± 0.98 ^a^	11.05 ± 0.38 ^b^	11.02 ± 3.84 ^a^	5.01 ± 0.99 ^b^	9.77 ± 2.20 ^b^	12.58 ± 1.40 ^a^	3.54 ± 0.18 ^bc^	6.34 ± 0.95 ^c^	7.51 ± 0.92 ^b^	2.54 ± 0.55 ^c^	3.64 ± 0.97 ^c^
5	4-Thujanol	546-79-2	0.59	0.22 ± 0.04 ^a^	0.13 ± 0.01 ^a^	0.23 ± 0.05 ^a^	0.25 ± 0.04 ^ab^	0.21 ± 0.00 ^a^	0.19 ± 0.01 ^a^	0.18 ± 0.02 ^bc^	0.17 ± 0.01 ^b^	0.17 ± 0.01 ^b^	0.14 ± 0.01 ^c^	0.12 ± 0.01 ^c^	0.12 ± 0.01 ^c^
6	Linalool	78-70-6	0.006	2418.58 ± 565.92 ^a^	2210.00 ± 368.84 ^a^	2136.67 ± 187.64 ^a^	2021.67 ± 326.70 ^a^	1949.46 ± 178.75 ^a^	1815.33 ± 203.00 ^b^	1731.34 ± 274.53 ^ab^	1758.67 ± 305.27 ^ab^	1466.62 ± 110.18 ^bc^	1443.3 ± 86.66 ^b^	1671.98 ± 36.83 ^b^	1639.33 ± 156.08 ^c^
7	Terpinen-4-ol	20126-76-5	1.2	0.59 ± 0.11 ^a^	0.50 ± 0.05 ^a^	0.43 ± 0.05 ^a^	0.47 ± 0.07 ^ab^	0.38 ± 0.05 ^b^	0.31 ± 0.01 ^b^	0.35 ± 0.03 ^bc^	0.28 ± 0.03 ^c^	0.22 ± 0.02 ^c^	0.21 ± 0.02 ^c^	0.21 ± 0.04 ^c^	0.15 ± 0.01 ^d^
8	α-Terpineol	98-55-5	1.2	1.44 ± 0.14 ^a^	1.13 ± 0.21 ^a^	0.85 ± 0.07 ^a^	1.14 ± 0.28 ^a^	0.98 ± 0.10 ^a^	0.73 ± 0.07 ^a^	0.52 ± 0.15 ^b^	0.63 ± 0.06 ^b^	0.58 ± 0.04 ^b^	0.23 ± 0.03 ^b^	0.30 ± 0.08 ^c^	0.26 ± 0.04 ^c^
9	α-Elemol	639-99-6	0.10	0.85 ± 0.19 ^a^	0.60 ± 0.04 ^a^	0.48 ± 0.03 ^a^	0.68 ± 0.10 ^ab^	0.53 ± 0.02 ^b^	0.47 ± 0.02 ^a^	0.43 ± 0.04 ^bc^	0.39 ± 0.02 ^c^	0.41 ± 0.01 ^b^	0.34 ± 0.02 ^c^	0.29 ± 0.01 ^d^	0.26 ± 0.02 ^c^
10	Nerolidol	40716-66-3	0.25	0.25 ± 0.05 ^a^	0.20 ± 0.01 ^a^	0.15 ± 0.01 ^a^	0.18 ± 0.03 ^b^	0.17 ± 0.01 ^b^	0.14 ± 0.01 ^b^	0.17 ± 0.02 ^b^	0.16 ± 0.01 ^b^	0.13 ± 0.00 ^b^	0.13 ± 0.01 ^b^	0.11 ± 0.00 ^c^	0.11 ± 0.01 ^c^
11	Tetradecane	629-59-4	1.00	0.52 ± 0.09 ^a^	0.40 ± 0.01 ^a^	0.33 ± 0.02 ^a^	0.34 ± 0.05 ^b^	0.29 ± 0.03 ^b^	0.32 ± 0.03 ^a^	0.19 ± 0.03 ^c^	0.22 ± 0.03 ^c^	0.26 ± 0.01 ^b^	0.17 ± 0.01 ^c^	0.18 ± 0.03 ^c^	0.15 ± 0.01 ^c^
12	α-Pinene	80-56-8	0.041	26.51 ± 5.71 ^a^	19.03 ± 0.96 ^a^	16.47 ± 1.54 ^a^	15.64 ± 1.97 ^b^	14.91 ± 3.12 ^b^	13.94 ± 1.73 ^a^	7.36 ± 1.72 ^c^	7.70 ± 0.96 ^c^	7.23 ± 0.91 ^b^	3.12 ± 2.04 ^c^	3.91 ± 0.66 ^c^	4.26 ± 1.27 ^b^
13	Sabinene	3387-41-5	0.016	436.59 ± 90.56 ^a^	378.23 ± 21.40 ^a^	307.87 ± 15.30 ^a^	212.16 ± 51.45 ^b^	208.16 ± 1.85 ^b^	162.92 ± 9.18 ^b^	105.89 ± 29.62 ^c^	110.84 ± 6.11 ^c^	102.80 ± 3.50 ^c^	43.83 ± 4.62 ^c^	73.68 ± 3.54 ^d^	61.06 ± 14.21 ^d^
14	β-Myrcene	123-35-3	0.0049	843.87 ± 158.49 ^a^	627.50 ± 46.47 ^a^	575.10 ± 30.81 ^a^	493.49 ± 72.56 ^b^	439.96 ± 6.99 ^b^	420.91 ± 48.23 ^b^	270.07 ± 35.42 ^c^	298.58 ± 14.16 ^c^	277.53 ± 20.45 ^bc^	256.03 ± 18.70 ^c^	278.67 ± 47.60 ^c^	276.98 ± 43.16 ^c^
15	γ-Terpinene	99-85-4	1.00	0.49 ± 0.09 ^a^	0.36 ± 0.03 ^a^	0.25 ± 0.01 ^a^	0.28 ± 0.04 ^b^	0.18 ± 0.00 ^b^	0.16 ± 0.01 ^b^	0.16 ± 0.02 ^b^	0.14 ± 0.01 ^c^	0.13 ± 0.00 ^c^	0.14 ± 0.04 ^b^	0.13 ± 0.01 ^d^	0.12 ± 0.01 ^d^
16	Terinolene	586-62-9	0.20	0.76 ± 0.14 ^a^	0.00 ± 0.00 ^a^	0.00 ± 0.00 ^a^	0.32 ± 0.05 ^b^	0.48 ± 0.01 ^b^	0.43 ± 0.03 ^b^	0.31 ± 0.04 ^bc^	0.30 ± 0.02 ^c^	0.18 ± 0.01 ^c^	0.15 ± 0.01 ^c^	0.16 ± 0.01 ^c^	0.16 ± 0.01 ^c^
17	β-Ocimene	13877-91-3	0.034	26.52 ± 7.22 ^a^	20.71 ± 2.14 ^a^	18.64 ± 1.63 ^a^	11.9 ± 2.16 ^b^	15.40 ± 0.37 ^b^	15.12 ± 1.47 ^b^	6.59 ± 1.13 ^b^	8.59 ± 0.41 ^c^	8.71 ± 0.25 ^b^	3.85 ± 0.30 ^b^	4.97 ± 1.06 ^d^	4.93 ± 0.77 ^c^
18	D-Limonene	5989-27-5	0.034	334.77 ± 49.40 ^a^	301.15 ± 15.27 ^a^	227.86 ± 15.42 ^a^	212.16 ± 51.36 ^b^	208.16 ± 31.09 ^ab^	162.92 ± 21.45 ^b^	105.89 ± 7.19 ^c^	110.84 ± 36.66 ^bc^	102.80 ± 39.74 ^b^	43.83 ± 1.49 ^c^	73.68 ± 55.29 ^c^	61.06 ± 8.45 ^c^
19	β-Caryophyllene	87-44-5	0.064	23.87 ± 3.70 ^a^	19.14 ± 1.98 ^a^	16.90 ± 1.14 ^a^	19.49 ± 2.28 ^a^	20.17 ± 0.94 ^a^	16.76 ± 2.01 ^a^	18.36 ± 2.13 ^a^	17.41 ± 1.03 ^ab^	15.77 ± 0.88 ^a^	16.98 ± 2.44 ^a^	14.77 ± 1.21 ^b^	15.07 ± 1.49 ^a^
20	α-Humulene	6753-98-6	0.16	4.38 ± 0.79 ^a^	5.91 ± 1.53 ^a^	3.44 ± 0.43 ^a^	2.88 ± 0.34 ^b^	3.87 ± 0.23 ^b^	2.03 ± 0.24 ^b^	2.18 ± 0.68 ^bc^	2.62 ± 0.12 ^b^	1.88 ± 0.13 ^b^	1.56 ± 0.21 ^c^	2.13 ± 0.27 ^b^	1.7 ± 0.19 ^b^
21	Caryophylene oxide	1139-30-6	0.41	1.28 ± 0.24 ^a^	0.90 ± 0.24 ^a^	0.65 ± 0.03 ^a^	0.84 ± 0.12 ^b^	0.54 ± 0.11 ^b^	0.49 ± 0.14 ^a^	0.48 ± 0.05 ^b^	0.51 ± 0.02 ^b^	0.35 ± 0.01 ^a^	0.03 ± 0.00 ^b^	0.37 ± 0.08 ^b^	0.34 ± 0.03 ^a^
22	Linalyl acetate	115-95-7	1.00	4.96 ± 0.92 ^a^	3.80 ± 0.30 ^a^	3.24 ± 0.23 ^a^	3.00 ± 0.45 ^b^	2.56 ± 0.06 ^b^	2.41 ± 0.33 ^b^	2.13 ± 0.28 ^bc^	2.09 ± 0.09 ^c^	1.74 ± 0.43 ^bc^	1.24 ± 0.10 ^c^	1.34 ± 0.23 ^d^	1.01 ± 0.29 ^c^
23	Phenethyl acetate	103-45-7	0.24959	0.22 ± 0.04 ^a^	0.13 ± 0.01 ^a^	0.20 ± 0.01 ^a^	0.14 ± 0.02 ^b^	0.12 ± 0.01 ^a^	0.14 ± 0.01 ^b^	0.03 ± 0.00 ^c^	0.06 ± 0.01 ^b^	0.12 ± 0.01 ^c^	0.00 ± 0.00 ^c^	0.05 ± 0.00 ^b^	0.05 ± 0.01 ^d^
24	Neryl acetate	141-12-8	0.042	3.01 ± 0.53 ^a^	2.08 ± 0.17 ^a^	2.04 ± 0.11 ^a^	1.44 ± 0.22 ^b^	1.83 ± 0.19 ^ab^	1.83 ± 0.10 ^a^	0.93 ± 0.15 ^b^	1.69 ± 0.08 ^b^	1.41 ± 0.10 ^b^	0.95 ± 0.06 ^b^	1.28 ± 0.06 ^c^	1.18 ± 0.12 ^b^
25	Geranyl acetate	105-87-3	0.15	1.13 ± 0.20 ^a^	0.00 ± 0.00 ^a^	0.00 ± 0.00 ^a^	0.95 ± 0.14 ^a^	0.00 ± 0.00 ^b^	0.00 ± 0.00 ^b^	0.40 ± 0.03 ^b^	0.32 ± 0.02 ^c^	0.38 ± 0.01 ^c^	0.36 ± 0.03 ^b^	0.30 ± 0.01 ^c^	0.34 ± 0.03 ^c^
26	β-Thujone	471-15-8	0.04	16.48 ± 2.63 ^a^	11.92 ± 0.90 ^a^	10.17 ± 1.07 ^a^	7.46 ± 2.03 ^b^	9.05 ± 0.34 ^b^	8.88 ± 0.51 ^a^	6.00 ± 1.59 ^b^	6.10 ± 0.25 ^c^	7.29 ± 0.42 ^b^	3.41 ± 0.85 ^b^	4.60 ± 0.37 ^d^	5.52 ± 0.20 ^c^

SS (SPI and MD) and SG (GA and MD) are the spray-dried microcapsules, respectively. Values represent the means ± standard deviation; *n* = 3. Different letters (^a^, ^b^, ^c^, ^d^) in each column indicate significant differences (*p* < 0.05).

## Data Availability

The data used to support the findings of this study can be made available by the corresponding author upon request.

## Supplementary Materials

The following supporting information can be downloaded at: https://www.mdpi.com/article/10.3390/foods13111726/s1. Figure S1: Changes in the total amount of six volatile compounds in PPO, SS, and SG within 15 days.
