# Supplementary figures and images for: Preparation and Characterization of Prickly Ash Peel Oleoresin Microcapsules and Flavor Retention Analysis
Source: Foods. 2024 May 31;13(11):1726. doi: 10.3390/foods13111726 (PMC11171865; doi:10.3390/foods13111726)

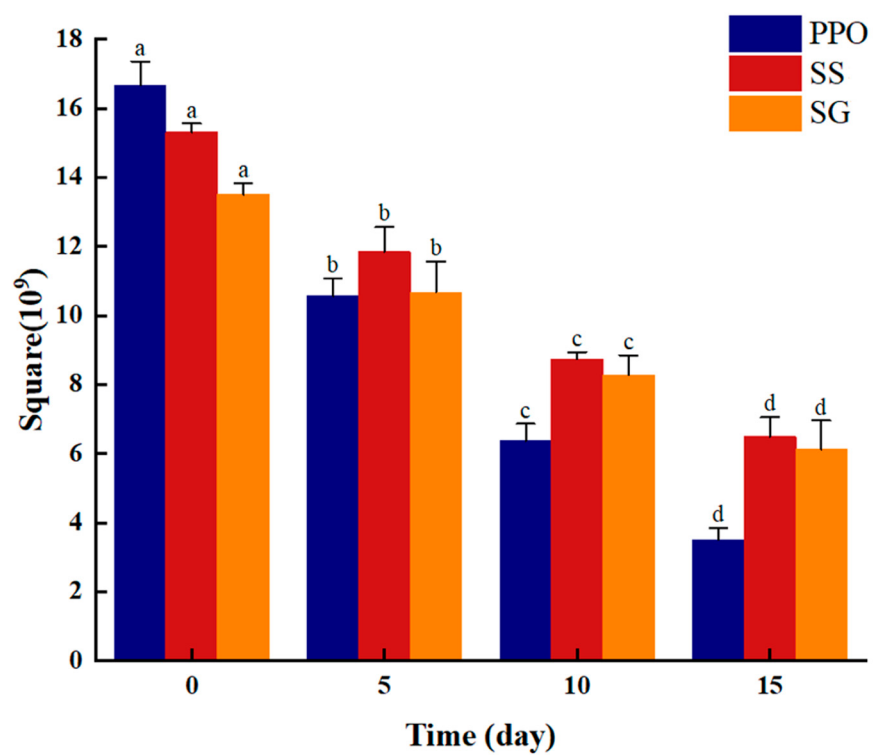

**Figure S1.** Changes in the total amount of six volatile compounds in PPO, SS and SG within 15 days.

Supplement: Supplementary file 1 [file foods-13-01726-s001.zip › foods-3013638-supplementary.pdf]
